# Celery Root Phenols Content, Antioxidant Capacities and Their Correlations after Osmotic Dehydration in Molasses

**DOI:** 10.3390/foods11131945

**Published:** 2022-06-30

**Authors:** Milica Nićetin, Lato Pezo, Marija Pergal, Biljana Lončar, Vladimir Filipović, Violeta Knežević, Hande Demir, Jelena Filipović, Dragan Manojlović

**Affiliations:** 1 Faculty of Technology Novi Sad, University of Novi Sad, Bul. Cara Lazara 1, 21000 Novi Sad, Serbia; cbiljana@uns.ac.rs (B.L.); vladaf@uns.ac.rs (V.F.); ovioleta@uns.ac.rs (V.K.); 2 Institute of General and Physical Chemistry, University of Belgrade, Studentski Trg. 12–16, 11000 Beograd, Serbia; latopezo@yahoo.co.uk; 3 Institute of Chemistry, Technology and Metallurgy, University of Belgrade, Njegoševa 12, 11000 Belgrade, Serbia; marijav@chem.bg.ac.rs; 4 Faculty of Engineering, Osmaniye Korkut Ata University, Osmaniye 80000, Turkey; hande.genckal@gmail.com; 5 Institute of Food Technology, University of Novi Sad, Bul. Cara Lazara 1, 21000 Novi Sad, Serbia; jelena.filipovic@fins.uns.ac.rs; 6 Faculty of Chemistry, University of Belgrade, Studentski Trg. 12–16, 11000 Belgrade, Serbia; manojlo@chem.bg.ac.rs; 7 South Ural State University, 76, Lenin Prospekt, 454080 Chelyabinsk, Russia

**Keywords:** antioxidant capacity, phenolic compounds, osmotic dehydration, celery root, sugar beet molasses

## Abstract

The osmotic dehydration (OD) of celery root in sugar beet molasses was studied at three temperatures (20, 35, and 50 °C) and three immersion periods (1, 3, and 5 h) in order to examine the changes in antioxidant potential and phenolic profile of celery root throughout the process. The antioxidant capacity (AOC) of dehydrated samples was evaluated by spectrophotometric and polarographic assays, the total phenolic content by the Folin-Ciocalteu method, and the individual phenolic compounds by HPLC-DAD. As a result of OD in molasses, the AOC and phenols content in samples increased proportionally to the augmentation of temperature and the immersion time. Vanillic acid, syringic acid, and catechin were detected in dehydrated samples as a result of transfer from molasses. Compared to fresh celery root, the content of identified phenols in osmodehydrated samples was improved from 1.5 to 6.2 times. Strong correlations between applied assays were obtained, except for the DPPH. Based on the correlation analysis chlorogenic acid, gallic acid, chrysin, catechin, and kaempferol showed the greatest contribution to the overall AOC of osmodehydrated celery root. Molasses, an agro-industrial waste from sugar production, could be valorized as a valuable osmotic solution.

## 1. Introduction

Celery (*Apium graveolens*) is one of the most important vegetables in the human diet and a medicinal herb in traditional medicine and pharmacology. Celery’s nutritional and therapeutic relevance derives from minerals, vitamins, dietary fiber, ß-carotene, essential oils, and phenols broadly present in this plant [1,2]. These functional components contribute to the overall antioxidant potential of celery leaves and roots. However, the phenols (flavonoids and phenolic acids) are the most responsible for celery’s pronounced antioxidant capacity (AOC) [3]. Many studies have verified that phenolic compounds are involved in preventing cardiovascular, neurodegenerative, and oncological diseases and also exhibit anti-inflammatory and antimicrobial properties [4,5].

In order to avoid the use of harmful synthetic antioxidants, as well as to find alternatives to the reuse of agricultural waste, special attention is paid to by-products of food industries, such as apple peels, citrus seed and peels, rice hulls, and sugar beet molasses, as sources of natural antioxidants [6].

Since phenols are unstable, i.e., very susceptible to degradation (by heating) or reaction with some factors (e.g., oxygen) during processing, which may result in a change in their structure and reduction of bioactive potential, the osmotic dehydration (OD) performed at ambient or lower temperatures can be considered one of the best preservation methods for maintaining antioxidant properties of food [7]. During OD, water is transferred through osmosis to the hypertonic solution in which it is immersed, whereas the solids from the osmotic solution are transported into the raw material [8]. Because the water is removed in a liquid phase, this method does not require energy for the latent heat of evaporation, leading to higher energy efficiency than the traditional drying process. Filipović et al. (2022) reported that by using osmotic dehydration (a low-energy dehydration method) as a pre-treatment, the lyophilization process (a high-energy dehydration method) may be shortened without compromising end product quality [7,9]. Sugar beet molasses, the thick, dark syrup that remains as agro-industrial waste after processing sugar beet into sugar, involved in OD as an osmotic solution, enables a good mass transfer during the process, favorable to water loss and at the same time for nutritional enrichment of osmotically dehydrated plant material [10]. Furthermore, because sugar beet molasses is a by-product of the sugar industry, using it as an osmotic solution is both cost-effective and environmentally friendly [11]. In addition to valuable nutritive content, molasses was confirmed as a promising source of residual antioxidants from the sugar beet (mainly phenolic compounds) and antioxidant components formed in sugar processing (mainly melanoidins) [12].

Different antioxidant components can exert variable behavior in various antioxidant assays, performed under different conditions, making comparing the results challenging [13]. Hence, insight into multifunctional behavior requires a multilateral approach, and evaluation of the AOC of the complex sample still demands the application of more than one method [14].

In previous work by Nićetin et al. (2021), the change in total AOC in celery leaves samples osmotically dehydrated in sugar beet molasses, as a function of process duration and temperature, was determined by various antioxidant assays, and the results showed that molasses influenced a slight increase in AOC in all analyzed samples [15]. This study analyzed the influence of sugar beet molasses during OD under different conditions on the content of identified phenolic compounds in celery root samples. The specific aim was to investigate the correlations between applied antioxidant assays, as well as between the content of individual phenolic compounds and the determined antioxidant activities in samples. Osmotically dehydrated celery root in sugar beet molasses can be potentially used for nutritional enrichment of various food products, such as biscuits, bread, snacks, yogurt, etc.

## 2. Materials and Methods

### 2.1. Material

The samples (roots of *Apium graveolens* L. var. rapaceum, Alabaster variety) were harvested at full maturity in October 2019, from individual producers, who cultivated them in the northern part of Serbia, the Province of Vojvodina.

Sugar beet molasses (provided from the sugar factory Crvenka, Serbia, with an initial dry matter content of 85.0%) was used as an osmotic solution. The dry matter content was determined refractometrically using an Abbe refractometer (Carl Zeiss, Jena, Germany).

### 2.2. Osmotic Dehydration

Samples of fresh celery root cut into cubes (1 cm × 1 cm × 1 cm) were dipped in laboratory jars with molasses. The mass ratio of fresh celery root samples and molasses was 1:20 in order to avoid excessive dilution of the molasses. Osmotic dehydration processes were performed at temperatures of 20 °C, 35 °C, and 50 °C, which were maintained constant in a heat chamber (Memmert IN 160, Schwabach, Germany). After immersion times of 1, 3, and 5 h, celery root samples were taken out from the molasses, lightly rinsed with distilled water, and gently blotted with paper to remove excess water [16].

### 2.3. Preparation of Celery Root Extracts

Fresh and osmotically dehydrated celery root samples were frozen and dried 24 h using an Alpha 1–2 (Christ, Osterode am Harz, Germany) freeze dryer. Dried samples were finely grounded into a powder using a Universal laboratory mill type WZ-1 (Spolem, ZBPP, Bydgoszcz, Poland) and passed through a 1 mm sieve. Two grams of powder for each sample were extracted with 200 mL of boiled water using a magnetic stirrer. After extraction, at room temperature for 10 min, the obtained aqueous extracts were filtered using Whatman No. 1 filter paper. The extracts were stored in a refrigerator (4 °C) until use in the AOC analysis [17].

### 2.4. Chemicals

Folin–Ciocalteu reagent, sodium carbonate, sodium acetate trihydrate, acetic acid, hydrochloric acid, potassium chloride, and sodium hydroxide were of analytical grade and were acquired from Merck, Darmstadt, Germany. 2,2-Diphenyl-1-picrylhydrazyl (DPPH) was produced by Fluka (Buchs, Switzerland), and methanol was obtained from J.T. Baker (Deventer, The Netherlands). 6-hydroxy-2,5,7,8-tetramethylchromane-2-carboxylic acid (Trolox), 2,4,6-tripyridyl-S-triazine (TPTZ), 2,2′-azino-bis (3-ethylbenzthiazoline-6-sulphonic acid (ABTS) diammonium salt), and gallic acid (GA) were purchased from Sigma-Aldrich Chemie (Steinheim, Germany). Hydrogen peroxide 35% (*v*/*v*) solution was medical grade (Belinka, Slovenia). Ethanol 96% (*v*/*v*) was obtained from Ada Vrenje (Belgrade, Serbia). Working standard solutions (2.0 mM) were prepared daily in ethanol or water.

Acetonitrile (>99.9%, Sigma-Aldrich HPLC grade), trifluoroacetic acid (>99.9%, Fisher Chemical analytical HPLC grade), and water (HPLC Plus grade water, Sigma-Aldrich) were used for HPLC analysis. Methanol Chromasolv gradient grade for HPLC was obtained from Sigma-Aldrich. Ethanol (96%) was purchased from Zorka Pharma, Šabac, Serbia. Hydrochloric acid (34–37%, TraceMetal Grade) was supplied from Fisher Chemical (Waltham, MA, USA). Syringe filters (25 mm, PTFE membrane 0.45 μm) were obtained from Agilent Technologies.

Apigenin (>95.0%, Sigma-Aldrich), luteolin (>99.0%, Fluka), quercetin (>95.0%, Sigma-Aldrich), chlorogenic acid (>98.0%, Sigma-Aldrich), rutin (>94.0%, Sigma-Aldrich), kaempherol (>90.0%, Sigma-Aldrich), galic acid (>98.0%, Fluka), caffeine (>99.0%, Sigma-Aldrich), chrysin (>96.0%, Sigma-Aldrich), *p*-coumaric acid (>98.0%, Sigma-Aldrich), caffeic acid (>98.0%, Sigma-Aldrich), vanillic acid (>97.0%, Fluka), synergic acid (>98.0%, Fluka), ferulic acid (>99.0%, Sigma-Aldrich), and catechin (>98.0%, Sigma-Aldrich) were used as standards.

### 2.5. Determination of Total Phenolic Content (TPC)

The content of phenols in the extracts was measured spectrophotometrically according to a modified method described by Gorjanović et al. (2012) with Folin–Ciocalteu’s (FC) reagent. Gallic acid was used as the standard, and the results were expressed as milligrams per liter of gallic acid equivalent (GAE) [18].

### 2.6. Antiradical Activity Determination by DPPH Assay

The antiradical activity of samples against DPPH radical (2,2-diphenyl-1-picryl-hydrazyl-hydrate) was determined by the method adopted from Brand-Willams et al. (1995). The AOC was expressed as millimoles Trolox equivalents/L using the calibration curve of Trolox (0–1000 μM), a water-soluble vitamin E analogue [19].

### 2.7. Determination of Total Reducing Power by (FRAP)

The FRAP assay (ferric-reducing antioxidant power) was carried out according to the standard procedure previously described [20]. Aqueous solutions of FeSO4·7H_2_O (100–1000 μM) were used for the calibration, and the results were expressed as millimoles per liter Fe (II).

### 2.8. Antiradical Activity Determination by ABTS Assay

The ABTS radical (2,2′-azinobis-(3-ethylbenzthiazolin-6-sulfonic acid)) was measured by the method adapted from Re et al. (1999) [21]. The results obtained from triplicate readings were expressed as Trolox equivalents and derived from a calibration curve determined from this standard (100–1000 μM).

### 2.9. DC Polarographic Measurements of AOC

Two direct current (DC) polarographic antioxidant assays, one based on a decrease of the anodic current of hydroxo perhydroxo mercury (II) complex (HPMC) formation in alkaline solutions of hydrogen peroxide at the potential of mercury oxidation, and the second based on a decrease of the DC polarographic cathodic current of mercury (II) reduction in the presence of antioxidants, i.e., mercury reduction antioxidant power (MRAP), were employed in parallel. The percentage of decrease of both currents was plotted against the volume of gradually added samples, and the slopes of these plots were used to express the AOC [18].

### 2.10. Identification and Quantification of Phenolic Compounds Using HPLC-DAD

Identification and quantification of phenolic compounds were done using high-performance liquid chromatography (HPLC) (Thermo Ultimate 3000 RS) with photodiode array detection (DAD) on a SupelcosilTM LC-18-DB analytical column (150 mm × 4.6 mm, 3 µm; Sigma-Aldrich) at 30 °C. The mobile phase consisted of 0.05% triflouracetic acid water solution as component A and 0.05% triflouracetic acid in acetonitrile as component B. The chromatographic elution was conducted at a flow rate of 1.0 mL/min in gradient mode: 0.0–7.0 min from 2% to 26% B, 7.0–8.0 min 26% B, 8.0–11.0 min from 26% to 2% B. The detector was set at 254 and 280 nm for the detection of phenolic compounds. The injection volume was 20 µL. Identification of the compounds was achieved by comparing their retention times and UV-Vis spectra with those of authenticated standards. Data analysis was performed with Chromeleon v6.8 software (Thermo Fisher Scientific, Bremen, Germany). All samples were analyzed in triplicate.

#### Sample Preparation for HPLC-DAD Analysis

The samples (2.0 g) were placed in a beaker (100 mL), and 60 mL acidic ethanol as a solvent was added and placed on Promax 2020 shaker (Heildoph, Germany). Extraction was performed using solvent containing 60% (*v*/*v*) ethanol and 1.2 mol/L HCl, with an extraction temperature of 50 °C and extraction time of 48 h. After that, the samples were ultrasonically extracted for 1 h at 40 kHz, 100 W in a Model Ultrasons 2.6 (Selecta, Spain). Finally, the extract was concentrated at 45 °C in a vacuum evaporator and analyzed by HPLC-DAD [22]. The obtained results were expressed as mg phenolic compound per kg dry matter of sample.

### 2.11. Statistical Analysis

The experimental data were derived from the full factorial (3 level, 2 parameter) experimental design with 9 samples. Principal component analysis (PCA) was applied to the exploratory data (descriptors) to characterize and separate the observed samples concerning the polyphenols’ profiles and the antioxidant assays. Results were expressed as mean ± standard deviation of triplicate analyses for all measurements. In addition, analysis of variance (ANOVA) for comparison of sample means was used to analyse variations in observed parameters among the samples.

Furthermore, to develop a rapid and accurate method for predicting the AOC based on phenolic content, the artificial neural network (ANN) model was developed, and its performance was evaluated on the experimental data. A multi-layer perceptron model (MLP) with three layers was used for modelling. The Broyden–Fletcher–Goldfarb–Shanno (BFGS) algorithm was used to solve the unconstrained nonlinear problems in the ANN modelling. The ANN experimental database was randomly divided into training, cross-validation, and testing data (with 60%, 20%, and 20% of the experimental data, respectively) [23].

The Yoon’s global sensitivity method was used to calculate the relative importance of the input parameters on output variables according to the weight coefficients of the developed ANN models [24].

The mathematical modelling consideration was performed using STATISTICA 10.0 (StatSoft Inc., Tulsa, OK, USA) [25].

## 3. Results and Discusion

In fresh celery root extract, 11 phenolic constituents were identified and quantified by HPLC-DAD (Table 1).

The major phenolics identified in the analyzed plant were four hydroxycinammic acids (*p*-coumaric acid, chlorogenic acid, ferulic acid and caffeic acid), one hydroxybenzoic acid (gallic acid), six different flavonoids components, three flavones (apigenin, luteolin, chrysin), and three flavonols (quercetin, rutin, and kaempferol). Compared to the other quantified phenolic compounds, flavonoid apigenin was the most abundant (700.32 mg/kg). According to the amount of flavonoids present, apigenin was followed by kaempferol, luteolin, chrysin, and quercetin. Among all detected phenolic acids, *p*-coumaric acid (81.54 mg/kg), caffeic acid (55.46 mg/kg), and ferulic acid (11.09 mg/kg) were prevalent in celery root extract. Yao et al. (2010) also stated that apigenin was the dominant flavonoid in celery leaves, followed by luteolin and kaempferol, and that *p*-coumaric acid was the dominant phenolic acid, followed by caffeic acid and ferulic acid [26]. This is in accordance with the presented results, but they could not detect chrysin and quercetin. Arsenov et al. (2021) reported that among phenolic acids, the predominant compound was ferulic acid, followed by chlorogenic and caffeic acid, and among the flavonoids, apigenin dominated in celery root [2].

In an extract of sugar beet molasses, the following 11 phenolic compounds were identified and quantified: apigenin, luteolin, kaempferol, chrysin, catechin, chlorogenic acid, gallic acid, *p*-coumaric acid, vanillic acid, ferulic acid and syringic acid (Table 1). Among the identified phenolic acids, ferulic acid (1560.66 mg/kg), vanillic acid (1260.76 mg/kg) and *p*-coumaric acid (1170.20 mg/kg) were prevalent. According to Chen et al. (2017), the major phenolics in sugar beet molasses included 10 components: gallic acid, p-hydroxybenzoic acid, vanillin, syringic acid, ferulic acid, catechin, cyanidin-3-*O*-rutinoside, cyanidin-3-*O*-glucoside, delfinidin-3-*O*-rutinoside, and delfinidin-3-*O*-glucoside [27]. Valli et al. (2012) reported that the most dominant phenols in sugar beet molasses were ferulic acid (14.83 μg/g), luteolin/kaempferol (17.24 μg/g), followed by syringic acid (2.26 μg/g) and vanillic acid (17.41 μg/g) [12]. The amounts of these same phenolics in this study were higher. The most abundant flavonoid was apigenin (1296.10 mg/kg), followed by kaempferol (980.34 mg/kg), catechin (860.74 mg/kg), and luteolin (402.55 mg/kg). There is no information in the literature about apigenin in sugar beet molasses, but it was detected in the extract of sugar cane [28].

As a result of OD of celery root in sugar beet molasses, all investigated phenolic compounds increased, except for quercetin, rutin, and caffeic acid, which could not be detected after OD. Devic et al. (2010) claimed that the main mechanism responsible for reducing phenolic compounds during the OD is water diffusion, because water-soluble phenols can be leached out with water flow from the plant material into the surrounding osmotic solution [29]. Another mechanism that may occur during OD and favors the loss of some individual phenolic is the hydrolysis of molecules. Hydrolysis of molecules reduces the degree of polymerization of some polyphenol compounds, resulting in molecules of lower molecular weight which diffuse more easily through the cell membrane into the surrounding solution [8]. These findings support the phenomenon that quercetin, rutin, and caffeic acid were already leached out from immersed celery tissue into molasses after an hour of the osmotic process.

On the other hand, the celery root samples treated in molasses showed higher responses on the chromatogram for apigenin, luteolin, kaempferol, chrysin, *p*-coumaric acid, chlorogenic acid, gallic acid, and ferulic acid in comparison to the chromatogram of the fresh celery root extract. Based on the results in Table 1, it is obvious that the increase in the content of these phenols was proportional to the increase of OD process parameters. After five hours of OD at the highest process temperature of 50 °C, in the dehydrated celery root sample, the amounts of phenolic compounds were the most improved: from 700.32 mg/kg to 1021.95 mg/kg for apigenin, from 69.54 mg/kg to 161.75 mg/kg for luteolin, from 4.50 mg/kg to 27.98 mg/kg for chlorogenic acid, from 600.26 mg/kg to 963.39 mg/kg for kaempferol, from 6.18 mg/kg to 10.94 mg/kg for gallic acid, from 198.81 mg/kg to 745.86 mg/kg for chrysin, from 81.54 mg/kg to 229.49 mg/kg for *p*-coumaric acid, and from 11.09 mg/kg to 39.14 mg/kg for ferulic acid. The largest increase in content compared to the initial state was recorded with chlorogenic acid (6.2 times), chrysin (3.7 times), and ferulic acid (3.5 times). It may be inferred that sugar beet molasses as a rich source of natural phenols improved the amounts of phenolic compounds present in celery root. An even more obvious proof of the positive effect of molasses on the phenolic profile of celery root was the detection of two hydroxybenzoic acids, vanillic acid and syringic acid, and one flavanol catechin in osmotically-treated samples. In fresh celery root extract, these three phenolic compounds have not been identified but appear in dehydrated samples due to diffusion from molasses during the OD. At higher process temperatures and by prolonging the immersion time, the permeability of cell membranes increased, and the viscosity of the sugar beet molasses decreased, causing mass transfer of phenolic compounds from molasses faster and easier [29]. Therefore, the content of vanillic acid, syringic acid, and catechin was increased with increasing temperature and duration of the process, from 6.78 mg/kg to 14.62 mg/kg, from 21.43 mg/kg to 38.78 mg/kg, and from 16.18 mg/kg to 28.65 mg/kg, respectively.

Additionally, the AOC value determined by FRAP, ABTS, and MRAP and the FRAP and TPC value were increased after OD in molasses for all observed experimental conditions (Table 2).

Nurkhoeriyati et al. (2021) reported that hot-air dried celery root slices compared with fresh samples had increased antioxidant activity but decreased total phenolic compound value [30].

In the work of Priecina et al. (2018), the total phenolic compound content in celery root samples increased using convective drying and microwave–vacuum drying, but flavonoid content decreased compared with fresh celery roots [31].

OD in molasses performed on the highest parameters (5 h and 50 °C) showed the largest enhancement in the FRAP, ABTS, DPPH, HPMC, and MRAP values compared to the fresh sample. The TPC in fresh celery root was 3.01 g GAE/L. A significant increase in total phenols was observed for all investigated samples osmotically dehydrated in molasses, in the range of 3.36–3.50 g GAE/L. Compared with this result, Salamatullah et al. (2021) reported a total phenol content of 2.2 g GAE/kg, Priecina and Karklina (2014) determined 3.3 g GAE/kg, while Golubkina et al. (2020) reported a TPC value of 10.8 g GAE/kg in celery roots [3,32,33]. The accumulation of phenols in a plant varies depending on the plant part and genotype, agroclimatic conditions, harvest time, and post-harvest processing [34]. The plants for this study were collected in their full maturity from the part of Serbia which is covered by agricultural land, the soil type is chernozem, and the climate is temperate continental, which are favorable conditions for celery production [2]. The post-harvest OD process in molasses further improved the examined celery’s antioxidant quality.

### 3.1. Correlation Analysis

Both HPMC and MRAP assays were applied in parallel with widely used spectrophotometric assays, FRAP, ABTS, and DPPH, to measure the AOC of examined celery root extracts, the TPC values of which were determined using the FC assay (Table 2). In addition, relative antioxidant capacity index (RACI), calculated by assigning equal weight to all AOC-applied assays (including TPC), were used to achieve a more comprehensive comparison between analyzed samples as well as applied assays [35]. Correlations between all these assays to determine the AOC of celery root extracts osmotically treated in sugar beet molasses are given in Table 3.

It was shown that the contribution of phenolics was predominant in the AOC determined by all assays applied but not by the DPPH scavenging activity. The correlation between HPMC and TPC was found to be very strict (0.90, *p ≤* 0.01), high between MRAP, ABTS, and TPC (0.82, 0.86, *p* ≤ 0.01), and somewhat lower but still statistically significant between FRAP and TPC (0.80, *p* ≤ 0.05). This behavior was widely demonstrated by many authors, who established a collectively-accepted fact that the antioxidant potential of different biological matrices mostly derived from the presence of phenolic compounds [4,35,36]. A complete lack of correlation between DPPH and TPC was observed, indicating that all other assays reflected the activity of a wider range of phenolics than DPPH. This could be because the DPPH method evaluates mainly the antioxidants of methanol extracts, and in this work, extracts were aqueous. Additionally, the explanation may be that the AOC is affected to a greater extent by the type of individual phenolic compounds than by the total phenolic content in the sample [14]. Moreover, DPPH was not correlated statistically significantly with any of the applied assays. This is in corroboration with the study by Fidrianny et al. (2013), who stated that there was no correlation between ABTS and DPPH scavenging activity in various extracts of sweet potato leaves [37]. Although DPPH and ABTS methods have the same mechanism of reaction based on the electron transfer, it was predicted that there was another different mechanism in these assays besides for electron transfer [4]. A distinct correlation between calculated RACI and TPC (0.88, *p* ≤ 0.01) confirmed an essential contribution of phenolic compounds to the total AOC. Furthermore, correlation analysis demonstrated that the RACI value correlated with each method.

In different methods for determining the AOC, the efficacy of antioxidant compounds varies due to the different chemical mechanisms behind the assays [14]. As seen, positively strong correlations exist between spectrophotometric assays (FRAP and ABTS) and slightly lower but still significant between polarographic ones (MRAP and HPMC). Additionally, both MRAP and HPMC correlated with ABTS and FRAP at a satisfactory level (0.79–0.92), indicating good agreement between DC polarographic assays and conventional spectrophotometric assays. Accordingly, the validity of polarographic assays in determining the AOC of celery root extracts was unequivocally confirmed.

In order to gain insight into the contribution of individual phenolic compounds to the total AOC determined by applied methods (FRAP, ABTS, DPPH, HPMC, MRAP, and TPC), correlation coefficients were calculated between their content obtained by HPLC-DAD analysis and the AOC of samples (Table 4).

In this context, there was an ability to perceive the possible relationships between the antioxidant activities and phenolic compounds. Correlations of applied antioxidant assays and individual phenolic content guided the main contributors of total AOC in samples [38]. Using Pearson’s correlation coefficient, which quantifies the association between two quantitative variables, it was possible to estimate the relationships between the different antioxidant activities of the extracts and their contents of phenolic compounds. Among all hydroxycinnamic phenolic acids present, chlorogenic acid had the most significant contribution to the total antioxidant activity determined by FRAP, ABTS, HPMC, and MRAP assays (0.94, 0.91, 0.87, 0.84, respectively, all statistically significant at the *p* ≤ 0.01 level). These results are in agreement with Zlatanović (2019), who states that chlorogenic acid content quantified in apple pomace flour correlated with ABTS (0.86) and HPMC (0.80) methods [39]. The hydroxybenzoic acid that showed the highest correlation with FRAP, ABTS, HPMC, and MRAP assays was gallic acid. A high to moderate correlation was also found with the remaining two hydroxycinnamic acids quantified in treated celery root extracts, namely, *p*-coumaric acid and ferulic acid. In addition, a moderate correlation between the content of vanillic acid and syringic acid derived from molasses, and the results of antioxidant assays was observed. In terms of flavonoids, flavon chrysin was correlated very well with the results of FRAP, ABTS, HPMC, and MRAP (0.87, 0.91, 0.76, and 0.93, respectively). Flavanol catechin, which diffused to the examined extracts from molasses, also showed excellent correlation coefficients with values of the AOC determined by these four assays (0.89, 0.943, 0.921, and 0.853, respectively). Additionally, catechin was associated with high AOC of apple by Plaza et al. (2014) [40]. However, a moderate correlation was noticed between the values of FRAP, ABTS, MRAP, and HPMC and the content of kaempferol and luteolin. On the other hand, apigenin, known as the main flavonoid in celery, which was the most abundant in the examined extracts of celery root, was less correlated with all applied assays, except for DPPH. This is in accordance with the study of Teixeira et al. (2017), where an inferior correlation between the flavonoid contents and the antioxidant activities of the extracts was observed [38]. They found that the correlation coefficient between the flavonoid content and the AOC as determined using the FRAP assay and ABTS scavenging assay were 0.47 and 0.49, respectively. As already noted, the DPPH method exhibited a different behavior compared to other assays employed in this study. According to correlation analysis, DPPH was positively correlated only with apigenin content, statistically significant at the *p* ≤ 0.05 level (0.80), and no statistically significant correlation was found with the other phenolic compounds. However, a moderate correlation was also observed between DPPH value and luteolin content (*r* = 0.56), which is the dominant flavonoid in celery root, besides for apigenin. Hirano et al. (2001) tested the radical scavenging capacity of various flavonoids and concluded that the most effective DPPH radical scavengers were: quercetin > catechin > kaempferol > luteolin > apigenin [41].

Based on high correlation coefficients (0.93, 0.89, 0.81, *p* ≤ 0.05, respectively), it was assumed that the content of gallic acid, catechin, and chrysin contributed the most to the TPC in examined extracts. In contrast, the three main representatives of flavonoids in celery root, namely, apigenin, luteolin, and kaempferol, showed the weakest correlation with TPC values (0.48, 0.65, and 0.60, respectively). These results suggest that besides for the flavonoids that are characteristic for celery root, other phenolic compounds such as phenolic acid or flavanols, from celery or derived from molasses, might make the principal contribution to the AOC of the examined extracts. The weaker correlations observed between the antioxidant activities and some phenolic compounds (such as apigenin) demonstrated that a considerable amount of these compounds in a plant does not always imply a corresponding antioxidant potential. RACI values were statistically significantly correlated with the content of all phenolic compounds present in the analyzed osmodehydrated celery root samples.

### 3.2. PCA Analysis

Principal component analysis is a mathematical procedure used as a central tool in exploratory data analysis [42]. PCA, applied to the given data set shown in Table 1 and Table 2, showed a differentiation between the samples according to the observed process parameters. Quality results showed that the first two principal components, accounting for 86.89% of the total variability for OD celery samples, can be considered sufficient for data representation.

**Figure 1:**
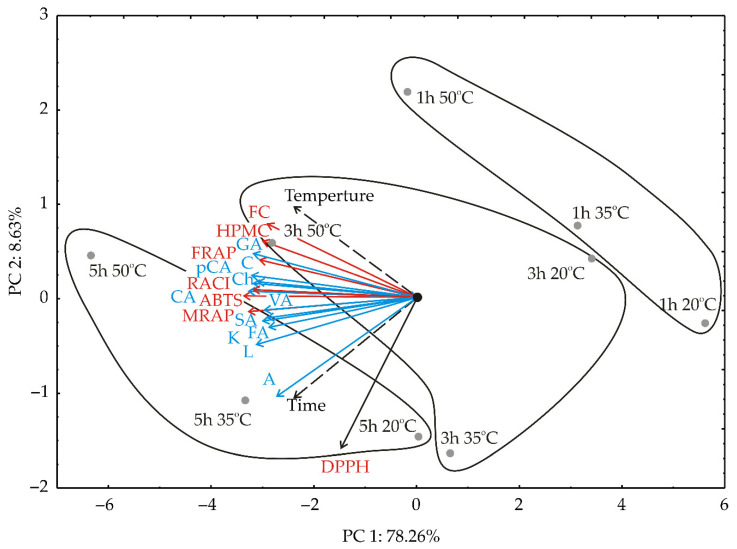
Biplot graphic of osmotically treated celery root samples.

Considering the map of the PCA performed on the data, the variables that contributed negatively according to the first principal component were: FRAP (which explained 5.8% of the total variance, based on the correlations), ABTS (6.6%), HPMC (5.5%), MRAP (6.6%), RACI (5.1%), TPC (6.9%), and the content of luteolin (6.0%), chlorogenic acid (6.2%), kaempferol (5.1%), gallic acid (6.1%), chrysin (6.2%), *p*-coumaric acid (5.8%), vanillic acid (5.3%), ferulic acid (5.1%), syringic acid (5.4%), and catechin (6.3%). The variables TPC (11.5% the total variance) and HPMC (6.9%) showed a positive impact on the second principal component, while the content of apigenin (19.1%) and DPPH (44.9%) showed a negative influence on the second principal component calculation (Figure 1).

According to PCA analysis, the first principal coordinate describes the difference between samples, based on variances in the time coordinate, while the second principal component shows the variations between samples caused by the temperature. The first group of osmotically dehydrated celery root samples (1 h 20 °C, 1 h 35 °C, and 3 h 20 °C) are located at the bottom right part of the graph. These samples were characterized as the samples being exposed to low temperature and a short time during OD, with lower AOC values. The second group consisted of samples 1 h 50 °C, 3 h 35 °C, and 5 h 20 °C, which were produced under a mild temperature regime and medium time. The highest AOC values were observed for samples 3 h 50 °C, 5 h 35 °C, and 5 h 50 °C, produced with the highest times and temperatures.

### 3.3. ANN Model

**Figure 2:**
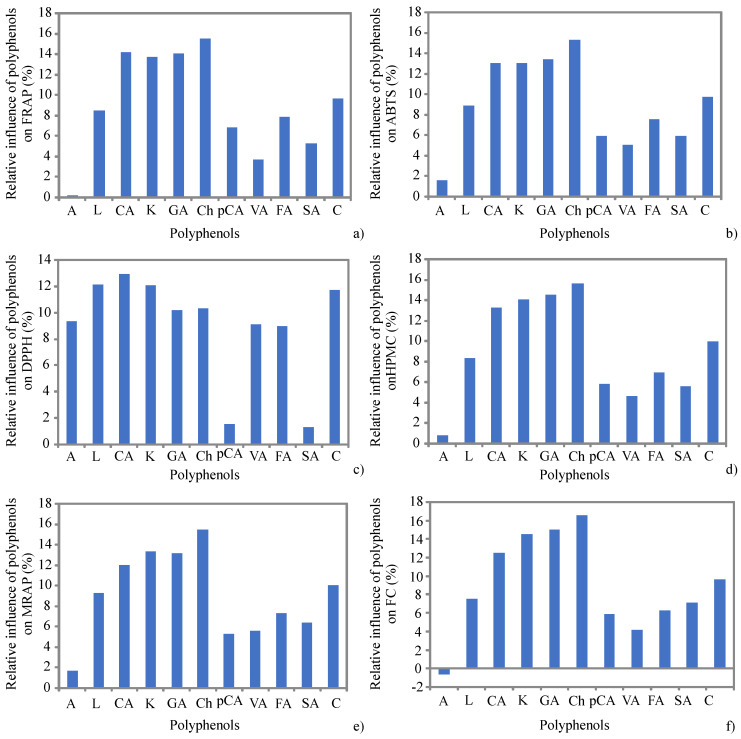
The relative importance of the content of apigenin (A), luteolin (L), chlorogenic acid (CA), kaempferol (K), gallic acid (GA), chrysin (Ch), *p*-coumaric acid (pCA), vanillic acid (VA), ferulic acid (FA), syringic acid (SA), and catechin (C) on FRAP—(**a**), ABTS—(**b**), DPPH—(**c**), HPMC—(**d**), MRAP—(**e**), and TPC—(**f**), determined using the Yoon interpretation method.

The influence of input variables (the content of apigenin, luteolin, chlorogenic acid, kaempferol, gallic acid, chrysin, *p*-coumaric acid, vanillic acid, ferulic acid, syringic acid, and catechin) on the antioxidant assays (FRAP, ABTS, DPPH, HPMC, MRAP, and TPC) during the OT were studied based on Yoon’s interpretation method of a developed ANN model. A graphical presentation of the ANN model results is shown in Figure 2.

According to ANN performance, the optimal number of neurons in the hidden layer for FRAP, ABTS, DPPH, HPMC, MRAP, and TPC calculations was 14 (network MLP 11–14-6) to obtain high values of coefficient of determination, *r^2^* (overall 0.812, for ANN during the training period), and low values of the sum of squares was 0.0005.

According to Figure 2, the most crucial influences on FRAP, ABTS, HPMC, MRAP, and TPC were observed for luteolin, chlorogenic acid, kaempferol, gallic acid, chrysin, and catechin contents. This conclusion was supported with the correlation analysis, where the correlation coefficients between antioxidant assays and the content of individual phenolic compounds were statistically significant. Additionally, PCA analysis showed a similar result, because the angles between antioxidant assays and the content of individual phenolic compounds indicated a high degree of their correlation (small angles corresponding to high correlations). The influences of *p*-coumaric acid, vanillic acid, ferulic acid, and syringic acid on FRAP, ABTS, HPMC, MRAP, and TPC can be interpreted as moderate. Additionally, moderate correlations were found between these phenolic acids and the values of the same antioxidant assays. However, apigenin showed slight impacts on FRAP, ABTS, HPMC, and MRAP, while on TPC, a negative influence was observed. On the other hand, a great impact of apigenin on DPPH was evident. Additionally, luteolin, chlorogenic acid, kaempferol, gallic acid, chrysin, vanillic acid, ferulic acid, and catechin had notable influences on DPPH.

## 4. Conclusions

This study indicated that osmotic dehydration in sugar beet molasses leads to improved phenol content and total antioxidant potential of celery root. By comparing the phenolic profiles of fresh and dehydrated celery root, it was observed that quercetin, rutin, and caffeic acid were lost due to the OD process. However, three phenolic compounds, which were not initially present, namely, vanillic acid, syringic acid, and catechin, were detected in dehydrated celery root samples through transfer from molasses during the process. As a result of OD in molasses, the content of apigenin, luteolin, chlorogenic acid, kaempferol, gallic acid, chrysin, *p*-coumaric acid, and ferulic acid, as well as AOC values (determined by FRAP, ABTS, DPPH, HPMC, and MRAP methods and TPC) in celery root gradually increased with increasing process parameters, as shown on the PCA biplot. These findings open a new perspective in which molasses, an agro-industrial waste from sugar production, could be transformed into a promising osmotic solution with the potential to enhance the antioxidant properties of food. Correlation analysis confirmed the similarity between all applied antioxidant assays, but the DPPH method showed different behavior. Contrary to expectations, apigenin, the most abundant phenol in celery, contributed the least to the AOC. The major contributors of total AOC of samples were chlorogenic acid, gallic acid, chrysin, kaempferol, and catechin, which were confirmed by correlation analysis and Yoon’s interpretation method of a developed ANN model. It can be concluded that celery root osmotically dehydrated in sugar beet molasses could be regarded as a valuable ingredient for various food formulations.

## Figures and Tables

**Table 1:** Contents of phenolic components in sugar beet molasses and fresh and osmotically dehydrated celery root.

Phenols (mg/kg)	Sugar Beet Molasses	Fresh Celery Root	Osmotically Dehydrated Celery Root in Sugar Beet Molasses								
			1 h 20 °C	1 h 35 °C	1 h 50 °C	3 h 20 °C	3 h 35 °C	3 h 50 °C	5 h 20 °C	5 h 35 °C	5 h 50 °C
Apigenin	1296.1 ± 64.85 ^d^	700.32 ± 35.04 ^a^	734.39 ± 36.49 ^a^	704.98 ± 34.88 ^a^	779.49 ± 38.59 ^b^	810.66 ± 40.93 ^b^	985.01 ± 49.78 ^c^	968.33 ± 49.31 ^c^	983.17 ± 48.51 ^c^	1004.28 ± 50.04 ^c^	1021.95 ± 50.24 ^c^
Luteolin	402.55 ± 20.05 ^g^	69.54 ± 3.47 ^a^	74.11 ± 3.67 ^a^	82.92 ± 4.21 ^b^	90.51 ± 4.61 ^b^	107.03 ± 5.25 ^c^	113.75 ± 5.78 ^c^	135.14 ± 6.84 ^d^	115.69 ± 5.85 ^c^	149.29 ± 7.48 ^e^	161.75 ± 8.04 ^f^
Quercetin	ND	12.34 ± 0.63	ND	ND	ND	ND	ND	ND	ND	ND	ND
Chlorogenic acid	368.24 ± 18.05 ^e^	4.50 ± 0.23 ^a^	5.55 ± 0.27 ^a^	14.76 ± 0.75 ^b,c^	15.64 ± 0.79 ^b,c^	12.86 ± 0.65 ^b^	17.59 ± 0.88 ^b,c^	19.85 ± 1.01 ^c^	14.61 ± 0.73 ^b,c^	19.13 ± 0.96 ^c^	27.98 ± 1.43 ^d^
Rutin	ND	23.61 ± 1.18	ND	ND	ND	ND	ND	ND	ND	ND	ND
Kaempferol	980.34 ± 48.44 ^e^	600.26 ± 30.59 ^a^	702.28 ± 34.91 ^b^	706.09 ± 34.80 ^b^	784.75 ± 39.90 ^c^	718.46 ± 35.89 ^b^	730.60 ± 37.04 ^b^	791.14 ± 39.94 ^c^	885.77 ± 44.87 ^d^	840.13 ± 42.44 ^cd^	963.39 ± 48.44 ^e^
Gallic acid	450.55 ± 22.26 ^b^	6.18 ± 0.31 ^a^	7.68 ± 0.39 ^a^	7.86 ± 0.39 ^a^	9.79 ± 0.48 ^a^	7.90 ± 0.39 ^a^	7.87 ± 0.40 ^a^	10.02 ± 0.50 ^a^	9.00 ± 0.44 ^a^	9.98 ± 0.49 ^a^	10.94 ± 0.55 ^a^
Chrysin	1907.12 ± 96.41 ^i^	198.81 ± 10.14 ^a^	221.78 ± 11.14 ^b^	416.73 ± 20.55 ^d^	591.40 ± 29.74 ^e^	381.24 ± 18.86 ^c^	421.32 ± 21.36 ^d^	618.32 ± 30.36 ^f^	626.95 ± 31.22 ^f^	636.19 ± 31.80 ^g^	745.86 ± 37.14 ^h^
*p*-Coumaric acid	1170.2 ± 59.26 ^i^	81.54 ± 4.11 ^a^	96.05 ± 4.76 ^b^	137.63 ± 7.01 ^d^	140.31 ± 7.00 ^d^	133.01 ± 6.76 ^d^	108.16 ± 5.38 ^c^	194.78 ± 9.87 ^g^	169.05 ± 8.51 ^e^	188.78 ± 9.42 ^f^	229.49 ± 11.45 ^h^
Caffeic acid	ND	55.46 ± 2.72	ND	ND	ND	ND	ND	ND	ND	ND	ND
Vanillic acid	1260.76 ± 64.25 ^f^	ND	6.78 ± 0.34 ^a^	9.40 ± 0.48 ^b^	10.32 ± 0.51 ^b^	7.42 ± 0.36 ^a^	11.72 ± 0.59 ^b,c^	11.93 ± 0.60 ^c^	7.63 ± 0.39 ^a^	15.25 ± 0.78 ^e^	14.62 ± 0.74 ^d^
Ferulic acid	1560.66 ± 77.19 ^i^	11.09 ± 0.55 ^a^	13.21 ± 0.66 ^b^	22.01 ± 1.12 ^e^	20.82 ± 1.05 ^d^	19.01 ± 0.96 ^c^	26.52 ± 1.35 ^f^	21.11 ± 1.04 ^de^	20.77 ± 1.06 ^d^	33.46 ± 1.69 ^g^	39.14 ± 1.95 ^h^
Syringic acid	472.69 ± 23.95 ^g^	ND	21.43 ± 1.06 ^b^	17.96 ± 0.90 ^a^	31.88 ± 1.60 ^d^	26.13 ± 1.30 ^c^	26.89 ± 1.33 ^c^	32.57 ± 1.63 ^d^	35.22 ± 1.75 ^e^	36.61 ± 1.84 ^ef^	38.78 ± 1.96 ^f^
Catechin	860.74 ± 43.14 ^h^	ND	16.18 ± 0.82 ^a^	19.54 ± 0.98 ^d^	22.58 ± 1.11 ^e^	17.69 ± 0.90 ^b^	22.17 ± 1.13 ^e^	27.74 ± 1.38 ^g^	18.85 ± 0.93 ^c^	25.65 ± 1.26 ^f^	28.65 ± 1.45 ^g^

Mean values in the same raw with different superscript are statistically different (*p* ≤ 0.05). ND—not detected.

**Table 2:** Change in antioxidant activity during osmotic treatment of celery root in sugar beet molasses.

Sample	FRAP (mM Fe(II)/L)	ABTS (mM TE/L)	DPPH (mM TE/L)	HPMC (%/mL)	MRAP (%/mL)	TPC (g GAE/L)	RACI
1 h 20 °C	1.542 ± 0.009 ^b^	1.104 ± 0.004 ^a^	0.469 ± 0.001 ^b^	0.043 ± 0.000 ^b^	0.020 ± 0.000 ^a^	3.360 ± 0.001 ^b^	−1.194
1 h 35 °C	1.560 ± 0.010 ^c^	1.116 ± 0.014 ^b^	0.465 ± 0.002 ^b^	0.042 ± 0.000 ^a^	0.021 ± 0.000 ^b^	3.374 ± 0.001 ^c^	−0.681
1 h 50 °C	1.562 ± 0.010 ^c^	1.122 ± 0.009 ^b^	0.462 ± 0.004 ^a^	0.043 ± 0.000 ^b^	0.022 ± 0.000 ^c^	3.498 ± 0.002 ^h^	0.1525
3 h 20 °C	1.550 ± 0.007 ^b^	1.110 ± 0.008 ^b^	0.461 ± 0.002 ^a^	0.042 ± 0.000 ^a^	0.021 ± 0.000 ^b^	3.385 ± 0.001 ^d^	−0.980
3 h 35 °C	1.558 ± 0.011 ^c^	1.120 ± 0.005 ^b^	0.479 ± 0.003 ^b,c^	0.042 ± 0.000 ^a^	0.022 ± 0.000 ^c^	3.396 ± 0.000 ^f^	0.0236
3 h 50 °C	1.571 ± 0.003 ^d^	1.129 ± 0.010 ^c^	0.473 ± 0.006 ^b,c^	0.043 ± 0.000 ^b^	0.022 ± 0.000 ^c^	3.502 ± 0.002 ^j^	0.8235
5 h 20 °C	1.560 ± 0.013 ^c^	1.118 ± 0.009 ^b^	0.476 ± 0.004 ^c^	0.042 ± 0.000 ^a^	0.021 ± 0.000 ^b^	3.390 ± 0.002 ^e^	−0.104
5 h 35 °C	1.560 ± 0.020 ^c^	1.130 ± 0.010 ^c^	0.477 ± 0.007 ^c^	0.043 ± 0.000 ^b^	0.022 ± 0.000 ^c^	3.468 ± 0.000 ^g^	0.6475
5 h 50 °C	1.580 ± 0.011 ^e^	1.132 ± 0.014 ^c^	0.473 ± 0.002 ^c^	0.044 ± 0.000 ^c^	0.022 ± 0.000 ^c^	3.502 ± 0.002 ^i^	1.314
Fresh	1.527 ± 0.016 ^a^	1.098 ± 0.011 ^a^	0.459 ± 0.000 ^a^	0.042 ± 0.000 ^a^	0.020 ± 0.000 ^a^	3.009 ± 0.001 ^a^	

Means in the same column with different superscript are statistically different (*p* ≤ 0.05). FRAP: ferric-reducing antioxidant power; ABTS: 2,2′-azinobis-(3-ethylbenzthiazolin-6-sulfonic acid); DPPH: 2,2-diphenyl-1-picryl-hydrazyl-hydrate; HPMC: hydroxo perhydroxo mercury (II) complex; MRAP: mercury reduction antioxidant power; TPC: Total Phenolic Content; RACI: relative antioxidant capacity index.

**Table 3:** Correlation coefficients between FRAP, ABTS, DPPH, HPMC, MRAP, TPC, and RACI for osmotically-treated celery roots.

	ABTS	DPPH	HPMC	MRAP	TPC	RACI
FRAP	0.898 **	0.256	0.891 **	0.787 *	0.797 *	0.913 **
ABTS		0.423	0.823 **	0.922 **	0.862 **	0.974 **
DPPH			0.155	0.460	0.634	0.466
HPMC				0.782 *	0.902 **	0.899 **
MRAP					0.818 **	0.941 **
TPC						0.876 **
RACI						

** Correlation statistically significant at *p* ≤ 0.01 level; * correlation statistically significant at the *p* ≤ 0.05 level.

**Table 4:** Correlation coefficients between antioxidant activities determined by applied assays and the content of individual phenolic compounds for osmotically dehydrated celery roots.

	FRAP	ABTS	DPPH	HPMC	MRAP	TPC	RACI
Apigenin	0.602	0.737 *	0.799 *	0.553	0.792 *	0.482	0.782 *
Luteolin	0.738 *	0.840 **	0.556	0.734 *	0.866 **	0.656	0.862 **
Chlorogenicacid	0.942 **	0.914 **	0.347	0.871 **	0.838 **	0.745 *	0.918 **
Kaempferol	0.737 *	0.716 *	0.393	0.673 *	0.876 **	0.604	0.788 *
Gallic acid	0.835 **	0.878 **	0.198	0.876 **	0.924 **	0.928 **	0.915 **
Chrysin	0.877 **	0.906 **	0.307	0.758 *	0.927 **	0.807 **	0.905 **
*p*-Coumaric acid	0.856 **	0.840 **	0.283	0.773 *	0.842 **	0.743 *	0.855 **
Vanillic acid	0.725 *	0.900 **	0.465	0.756 *	0.843 **	0.741 *	0.873 **
Ferulic acid	0.726 *	0.804 **	0.434	0.694 *	0.830 **	0.566	0.799 *
Syringic acid	0.663	0.783 *	0.418	0.687 *	0.929 **	0.743 *	0.833 **
Catechin	0.894 **	0.943 **	0.377	0.921 **	0.853 **	0.894 **	0.965 **

** Correlation statistically significant at *p* ≤ 0.01 level; * correlation statistically significant at *p* ≤ 0.05 level.

## Data Availability

Data are contained within the article.
